# Determination of Morphine in Human Urine by the Novel Competitive Fluorescence Immunoassay

**DOI:** 10.1155/2019/7826090

**Published:** 2019-02-03

**Authors:** Jie Cao, Xiao-Ying Chen, Wu-Rong Zhao

**Affiliations:** ^1^College of Life Sciences, Fujian Agriculture and Forestry University, Fuzhou 350002, China; ^2^Scientific Research and Experiment Center, Fujian Police College, Fuzhou 350007, China; ^3^AQSIQ Key Laboratory of Drug Detection, Fujian International Travel Healthcare Center, Fujian Entry–Exit Inspection and Quarantine Bureau of P.R.C., Fuzhou 350001, China; ^4^Fujian HuaMin Forensic Center, Quanzhou 362000, China; ^5^College of Environmental and Resource, Fuzhou University, Fuzhou 350116, China

## Abstract

A competitive fluorescence immunoassay for the identification and quantification of morphine has been developed on the basis of hapten-coated plate format. Hapten was prepared through covalent conjugating a morphine derivative with albumin bovine. In the immunoassay, the hapten was inoculated on a 96-well plate and then bound with monoclonal antibodies labeled with a signal indicating dye, fluorescein isothiocyanate (FITC). Unbound FITC-antibodies were rinsed off from the plate. The fluorescein intensity decreases in the presence of morphine molecules due to the competitively binding to antibodies against hapten. The intensity is inversely correlated with the concentration of morphine. In quantitative analysis for urine samples, we obtained a linearity range of 0.2 *μ*g/mL∼2.5 *μ*g/mL, along with a detection limit of c.a. 1 ng/mL. The fluorescence immunoassay shows low cross-reactivity (below 10%) to 6-acetylmorphine, 3-acetylmorphine, and heroine. The developed method produced comparable results to the standard GC-MS/MS method. In conclusion, a rapid and efficient screening tool for morphine in clinical human urine has been established.

## 1. Introduction

Morphine (MOR), which is one of the main components in opium, is widely used in relieving severe pain for patients, especially those undergoing a surgical operation, and it is recommended for mildly alleviating cancer-related pain by the World Health Organization (WHO) [1]. MOR is a precursor of narcotic analgesics, among which the most representative drug are heroin, codeine, pethidine, etc. MOR analysis in biological fluids has been applied in forensic cases as an indicator of heroin usage [2] and in routine pharmacokinetic studies [3]. Abused and overdosed of MOR drug is not only dangerous to human health but also injury to the central nervous system, especially the fatal ingested dose is 120 mg. It has been reported that almost 10% of discharged MOR remains unmetabolized, and 90% of orally administrated MOR is passed in the urine within 24-hour period [4]. Therefore, to prevent overdose-induced toxicity, it is very necessary to adopt precise examination means for sensitively monitoring the concentration of MOR in patients' blood and urine sample [5, 6].

In recent years, with the development of the theory and mechanical instruments, the analytical instrument for measuring MOR in urine was multifarious, such as gas chromatography (GC) [7], liquid chromatography (LC) [8–10], UV spectrophotometer [11], GC-mass spectrometer [12–15], LC-mass spectrometry [16–18], fluorimetry [19], chemiluminescence [20], surface plasmon resonance (SPR) [21], electrochemical methods [22–24], etc [25, 26]. However, some of these techniques are time-consuming, have low anti-interference ability, have large fluid sample usage, use expensive equipment, are toxic solvents, etc. In some cases, special pretreatment to the sample is required before analysis. Among them, immunoassays have received much interest in recent years for pharmaceutical determination due to their advantages of ease to use, portability, and disposability [27–30].

In this work, a competitive immunofluorescence method was used to qualitatively and quantitatively analyze MOR in human urine. For the competitive immunofluorescence method, fluorescein isothiocyanate (FITC) was used as the fluorescent dye to label antibodies. MOR in sample solution and the hapten coated on microporous plate competed with each other to bind with FITC-labeled MOR antibody. The fluorescence intensity is associated with the concentration of MOR, and the correlation index of the affine function for MOR was greater than 0.99%. The relative standard deviation (RSD) of the results obtained from the new method was lower than GC-MS/MS analysis results, while detection limit of our new method was two orders of magnitude lower. The results show that we have successful established a new competitive immunofluorescence method to detect MOR in human urine. Most importantly, the new method does not require sample preparation as other methods do.

## 2. Materials and Methods

### 2.1. Apparatus

The measurement of fluorescence was carried out by Varioskan Flash Multimode reader (Thermo Fisher Scientific, USA). 96-well plates were purchased from NUNC (Denmark). Hapten conjugation was verified via infrared spectrometer (Vertex 70, Bruker Optics, USA) and MALDI-TOF-MS spectrometer (autoflex speed, Bruker, USA). The acidity and alkalinity of all solutions were measured by Denver UB-10 UltraBasic meter (Denver Instruments, USA). Throughout the experiment, the immunization program was carried out in the constant temperature incubator with constant temperature control accuracy of 0.1°C.

### 2.2. Reagents

MOR standard was purchased from National Institutes for Food and Drug Control of China (Beijing, China). Bovine serum albumin (BSA) and ovalbumin (OVA) were purchased from Sigma-Aldrich (St. Louis, MO, USA).

The following buffer solutions were prepared: carbonate-buffered saline (CBS, 15 mmol/L·Na_2_CO_3_, 35 mmol/L·NaHCO_3_, pH 9.6), CBS buffer (1 mol/L·Na_2_CO_3_, 1 mol/L·NaHCO_3_, pH 8.9), phosphate-buffered saline (PBS, 8.5 mmol/L Na_2_HPO_4_, 1.5 mmol/L·KH_2_PO_4_, 137 mmol/L·NaCl, 2.7 mmol/L·KCl, pH 7.4) including equivalent to 10 mmol/L phosphate, and PBST buffer (PBS containing 0.05% Tween 20, pH 7.2). The stock solutions of MOR hapten were prepared in CBS buffer (pH 9.6). MOR antibodies labeled with FITC were dissolved into PBS buffer. PBST buffer was used as the 96-well plate cleaning solution. The blocking agent was the OVA solution that was contained 0.1% (v/v) OVA dissolved in PBS buffer.

Unless special instructions, the experimental data were obtained by averaging five groups parallel experiments. Throughout the experiment, all chemicals were analytical grade and redistilled water was used.

### 2.3. MOR Hapten Synthesis

**Figure 1:**
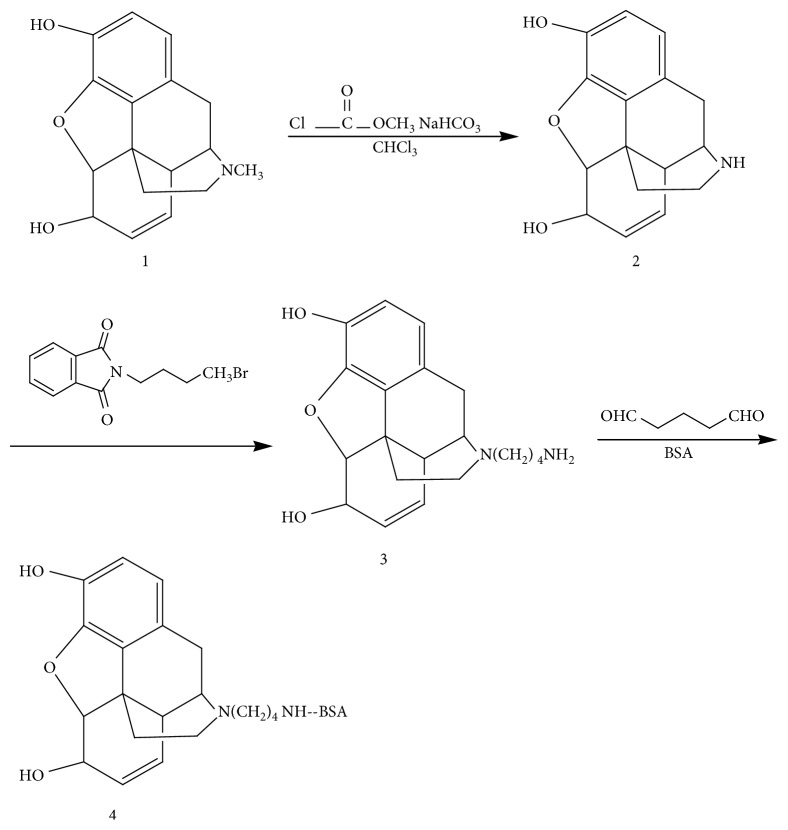
Synthesis reaction process of MOR hapten [31].

An amine-MOR hapten was synthesized according to the literature procedures (see Figure 1) [31]. In order to be immunogenic, the target MOR molecule with small relative molecular weight (MW 285) was conjugated with a carrier protein, forming complex biological macromolecules.

**Step 1 :**

synthesis of normorphine, as shown as Compound No. 2 in Figure 1. The detailed experimental procedure is as follows: MOR base (1 g), methyl chloroformate (5 mL), and sodium bicarbonate (4.2 g) were added into chloroform solution (50 mL). The reaction solution was refluxed under constant 68°C for 20 h and filtering and purifying the residue for several times with chloroform (5 mL). The removing water of the residue was performed by the dried sodium sulfate. At last, pure yellow foam (1.47 g) could be acquired after repeating the same operation several times. The foam was slowly dropwise added into 3 mL hydrazine hydrate (volume fraction *φ* is 0.97), then mixed with 4 ml of hydrazine hydrate (*φ* is 0.64), and refluxed for 63 h in an oil bath. Subsequently, cooling, crystallization, filtration, and washing with water, acetone, and chloroform three times, and red solid precipitate was obtained in 68.5% yield.

**Step 2 :**

synthesis of amine-functionalized normorphine, as shown as Compound No.3 in Figure 1. Compound 3 is N-4-aminobutyl normorphine. The detailed experimental procedure is as follows: Normorphine (0.3 g), N-(4-bromobutyl) phthalimide (0.5 g), and anhydrous sodium carbonate (0.5 g) were mixed together, placed into the three-neck round-bottom flask containing dimethylformamide (7 mL) and refluxed for 2 h, and the reaction solution was vacuum filtered to reduce the liquid concentration. 5 ml hydrazine hydrate (*φ* is 0.90) was putted into the reaction solution and refluxed for 1 h under nitrogen atmosphere. After vacuum filtration, the white compound was precipitated and washed with a small amount of water. Finally, drying naturally and recrystallized in methanol, the white precipitate had a 73.2% yield.

**Step 3 :**

synthesis of MOR-BSA conjugate, as shown as Compound No. 4 in Figure 1. The detailed experimental procedure is as follows: *N*-(4-aminobutyl) normorphine (20 mg) and BSA (20 mg) were dispersed in the double distilled water (20 mL), and the pH value of the mixture is adjusted to neutral with the aid of acid-base buffer, adding aqueous glutaraldehyde (2.2 mL) and stirred overnight at 4°C. The resulting MOR hapten was purified with a SephadexG-25 column, and the first peak wavelength of 280 nm was collected, concentrated, and lyophilized.

### 2.4. Verification of MOR Hapten

Firstly, structural identification of MOR hapten was analyzed by infrared spectroscopy. Lyophilized MOR hapten was mixed with dry potassium bromide, and the ground mixture was pressed into the pellet, and then the pellet was analyzed using the infrared spectrometer. Lyophilized BSA standards and MOR were also tested as references.

Secondly, structural identification of MOR hapten was analyzed by the MALDI-TOF MS (Bruker Daltonics). The MALDI-TOF MS can detect positively charged peptide ions. Automatic detection of the protein was implemented using the FlexAnalysis software (Bruker Daltonics). A mixture solution of 0.5 *μ*L BSA and 0.5 *μ*L sinapinic acid (10 mg/mL in 50 : 50 ACN/H_2_O 0.1% TFA) was spotted on a MALDI-TOF MTP 384 polished steel. The protein-matrix samples were allowed to crystallize at room temperature. Prior to measurements, the instrument was calibrated against a BSA MALDI-TOF calibration standard; therefore, the ionization of the MorHap-BSA (MOR hapten-BSA) conjugates was expected to be similar, and any mass shift was attributed to hapten incorporation into the protein.

### 2.5. MOR Antibody Preparation

The lymphatic subcutaneous tissue of 8-week-old female rat (BALB/c) was immunized. After centrifugation and precipitation, the celiac cell suspension was obtained and the good growing myeloma cells (SP2/0) status was obtained by subculture. Taking off the lymph node that has been immunologically treated, the cell culture medium IMDM containing lymphatic particles was obtained. Then the lymphocyte, myeloma cell, and celiac cell suspension were mixed together in proportion and fused by the polyethylene glycol (PEG) pure fusion method to obtain hybridoma cells. The hybridoma cell line 3E7 with the highest inhibition rate (100%) was prepared by competitive inhibition ELISA. The monoclonal antibody with positive rate up to 100% was purified by HiTrap PROTEING HP affinity column.

### 2.6. Conjugation of Fluorescein Isothiocyanate and Anti-MOR Monoclonal Antibody

**Figure 2:**
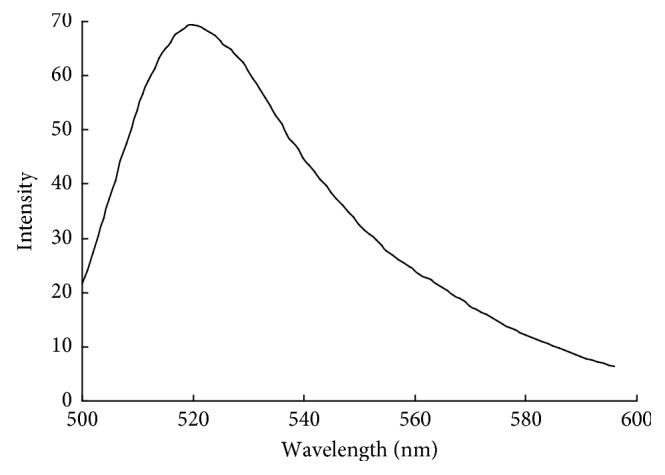
Fluorescence spectrum of FITC.

According to the method in the literature [32], anti-MOR monoclonal antibodies were conjugated with FITC. Anti-MOR monoclonal antibody was dispersed in PBS buffer, FITC was dissolved in the CBS buffer (pH 9.6), and the two are mixed together, stirring at constant 20°C for 4 hours. The resulting conjugates were dialyzed against PBS (pH 7.4) for 4 h and treated by Sephadex G-50 for filtration of the remaining free dye [33]. Then, the absorption of FTIC-labeled antibody was measured by using the UV spectrophotometer, shown in Figure 2. In comparison with the pure FITC standard, the concentration of FITC was estimated to be 1.105 *µ*g/mL and anti-MOR monoclonal antibody was 0.0886 mg/mL. The approximate molar ratio of FITC and anti-MOR monoclonal antibody (*n*_FITC_/*n*_MOR-Ab_) was estimated to be 8.1.

### 2.7. Fluorescence Immunoassay Process

The fluorescence immunoassay process was operated on the 96-well microtiter immunoassay plate. Throughout the experiment, PBST was used as the rinsing solution. By slow dropping, MOR hapten conjugation solution was coated to the surface of the orifice and incubated at 4°C for 12 h, nonspecific binding sites were blocked with OVA in PBS (350 *µ*L, 1%) at constant 37°C for 0.5 h, and then FITC (100 *µ*L) labeled anti-MOR monoclonal antibody (diluted in PBS) was mixed with sample solutions or various MOR standard solutions (known concentration, diluted in 0.01 mol/L PBS, pH 7.4). Then, the reaction was incubated at 37°C for 2.0 h, which are the optimum temperature and time. The plate was read by the automatic detection microplate reader being excited at 488 nm (*λ*_ex_) and recorded at 525 nm (*λ*_em_) (*λ*_ex_ = 488 nm, *λ*_em_ = 525 nm). *F*_max_ was defined as the fluorescence intensity measured in a MOR-free sample; *F* was defined as the fluorescence intensity measured in the presence of MOR in the sample; *F*_0_ was defined as the fluorescence intensity measured in a condition that no FITC-labeled antibody is available to bind to the surface-immobilized hapten because of the excessive MOR in the sample. The fluorescence intensity was plotted against the concentration of MOR, yielding to a competitive curve. All values are averaged from five parallel measurements. The half maximal inhibitory concentration (IC_50_ value) was detected by the analyte concentration at 50% of the maximal quenching. The limit of detection (LOD) is defined as three times over the background, the signal-to-noise ratio.

### 2.8. GC-MS/MS Analysis

The GC-MS method is a standard method in FDA China. Our lab is a certified testing lab. All the GC-MS testing followed the exact procedure as the standard method. The urine sample of MOR abusers was collected from Fujian HuaMin forensic center, a large forensic center in the province. Samples were sealed in Teflon centrifuge tubes, and sample amounts were greater than 5 mL. Sample pretreatment was conducted by first placing urine sample (1 mL) into the centrifuge tube (10 mL). 0.2 mL concentrated hydrochloric acid was dropped into urine sample and then heated by water-bath at 70°C for 30 min. Reaction solution was cooled, and 8 mol/L natrium hydroxydatum was added to adjust the pH to 9.0. CBS buffer (pH 8.9) solution (1 mL) was added and leached out by the mixture of chloroform and isopropanol (3 mL, volume ratio 9 : 1). The mixture was sonicated for 10 min, and then centrifuged at 10000 rpm for 3 min. This process was repeated twice, and the supernatant was collected and combined in a separate test tube. 5 mg anhydrous sodium sulfate was added. The supernatant fluid was transferred into another centrifuge tube and dried in nitrogen atmosphere at 40°C. The pellet in centrifuge tube was dissolved with the mixture of ethyl acetate (200 *μ*L) and derivatization reagents (150 *μ*L). The test tube was sealed with parafilm and underwent derivatization reaction for 30 min at 75°C. By centrifugation, filtration, and nitrogen drying at 40°C, final product was dissolved in ethyl acetate (200 *μ*L) and injected (1 *µ*L) into the GC-MS/MS for testing.

Chromatographic analysis was performed on an apparatus model Agilent 7890 A/7000QQQB equipped with HP-5 capillary column (30 m × 0.25 mm × 0.25 *μ*m). The initial oven temperature was 150°C and was maintained for 1 min; then the temperature was raised to 280°C at a rate of 60°C/min and maintained for 6 min, resulting in a total run time of 9.17 min. The carrier gas (He) was used, and the GC was operated in splitless mode at a constant flow rate of 1 mL/min. The temperature of the gasification chamber was 250°C. The mass spectrometer was equipped with EI source operated at 70 eV with source temperature of 230°C. The temperature of the tandem quadrupole was 150°C, and nitrogen was utilized as the collision gas. Mass spectra were monitored by the multiple reactions monitoring (MRM) acquisition mode. The MS/MS parameters of MOR were optimized by selecting unique precursor ions, product ions, and the collision energies (CE) that achieved the highest selectivity and maximum area counts. Three optimal MOR precursor-product transitions were 429-429.1 transition matching CE 5V, 429-287.2 transition matching CE 20V, and 429-146.1 transition matching CE 35V. The retention time of MOR was 5.311 min. The 429-429.1 transition of the most abundant mass in molecular ion cluster was chosen as the quantitation ion for MOR, and the remaining two transitions were qualifier ions.

## 3. Results and Discussion

### 3.1. Verification of MOR Hapten (MOR-BSA)

**Figure 3:**
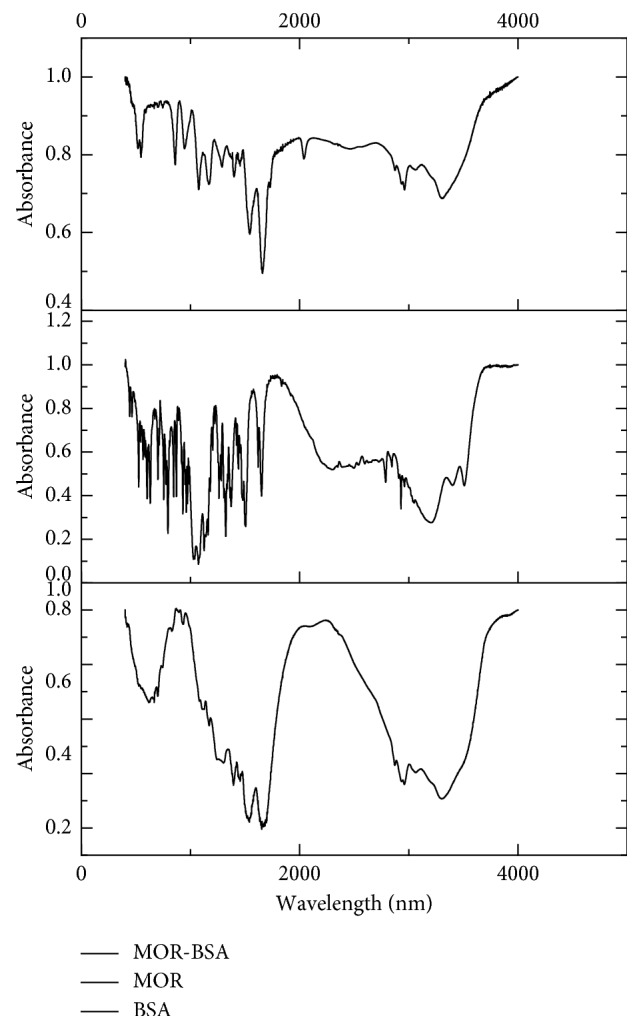
IR spectrum of MOR hapten.

Using solid potassium bromide tableting, BSA, MOR, and MOR-BSA were detected by infrared spectrometry. As seen on Figure 3, by comparing the infrared absorption spectrum of the BSA and MOR-BSA, we observed that the 2800–3400 cm^−1^ and 1500–1700 cm^−1^ areas had similar absorption which was characteristic of amino acids in the protein. These suggest that MOR-BSA contains characteristic functional groups found in BSA. Compared to the infrared spectrum of MOR, MOR-BSA did not contain the symmetric and antisymmetric stretching vibration of MOR (tertiary amine salt ions N^+^–H in 2538 cm^−1^, 2123 cm^−1^ and 2650 cm^−1^). Moreover, the stretching vibration of N–H in 3501 cm^−1^ was present. The infrared spectrum suggested that MOR-BSA was successfully synthesized.

**Figure 4:**
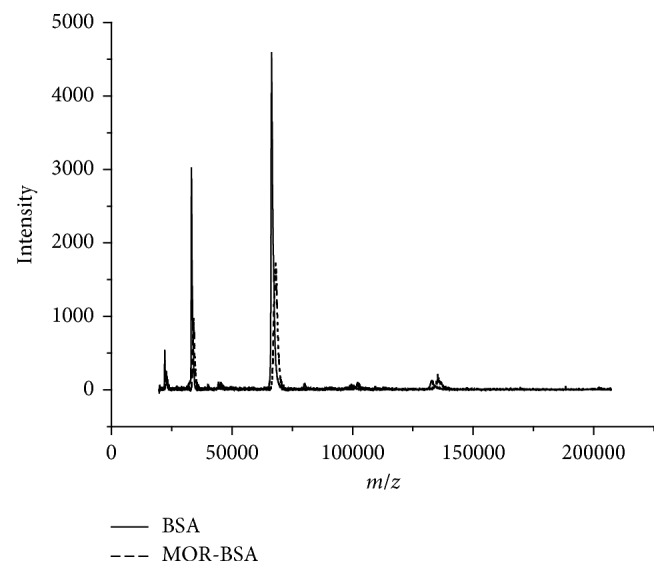
Spectrogram of MOR hapten by MALDI-TOF-MS method.

Synthesis of MOR-BSA was further verified by MALDI-TOF-MS. Compared to the mass spectrum peak of BSA (solid line) in Figure 4, that MOR-BSA (dotted line) was shifted to right. This indicated the molecular weight of MOR-BSA was larger than BSA, which further confirmed that MOR-BSA was synthesized successfully. Furthermore, Figure 4 shows that the molecular weight of MOR-BSA is 68467.3 and that BSA is 66304.5. Therefore, the numbers of BSA combining with MOR were average of six.

### 3.2. Fluorescence Immunoassay Optimization

With the improvement of analysis technology, the demand for large-amount analysis of small molecular substances such as drugs and hormones in clinical analysis experiments has never been larger than now. Different from biochemical test or enzyme analysis, immune analysis has gained credibility in its correctness and accuracy, due to its targeting and precision. As MOR structure is a small molecule structure, immune analysis must be performed under competitive mechanisms in order to be accurately tested. By coupling MOR with antibody-FITC, a new competitive fluorescence analysis method was used for recording the fluorescence intensity changes of MOR. FITC-labeled antibody mixed with sample solution or various MOR standard solutions was added into the MOR hapten-coated plates. In this immunoassay, the more MOR was mixed into the MOR antibody-FITC solution, the less MOR antibody-FITC can combine with MOR hapten coated in plates, and the lower the fluorescence intensity displayed. In order to monitor the low dosage of MOR, highly sensitive detection conditions were required, such as coating MOR hapten concentration, time and temperature of the hapten coated, pH value and ionic strength of buffer solution, incubation time, temperature of antigen-antibody reaction, etc.

### 3.3. Concentration of Coating MOR Hapten

**Figure 5:**
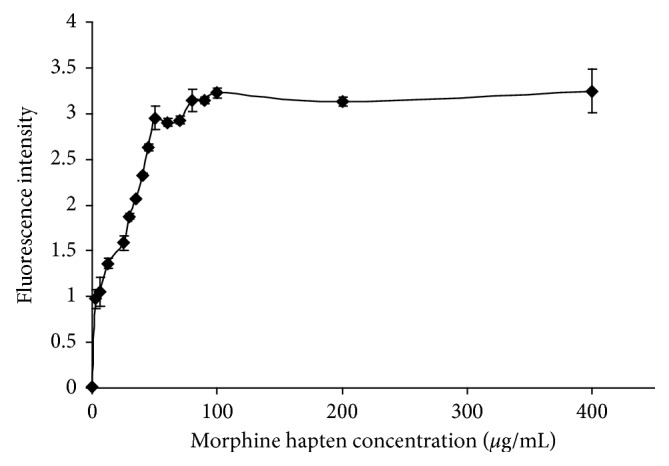
Optimized curve of MOR hapten concentration.

In order to select the optimal coated concentration of MOR hapten, different concentrations of MOR hapten diluted by CBS buffer (pH 9.6) solution were coated on microtiter plates. The equal quantity of anti-MOR monoclonal antibodies labeled with FITC was added into every well, and the fluorescence intensities were measured. Titration curves of different concentrations of coated MOR hapten (0.3, 3, 6.25, 12.5, 25, 30, 35, 40, 45, 50, 60, 70, 80, 90, 100, 200, and 400 *μ*g/mL, respectively), and corresponding acquired fluorescence intensity, which is the average value of five times' measurements, were made. As shown in Figure 5, the corresponding fluorescence intensity immediately increased progressively as soon as the concentration of coated MOR hapten was increased and reached a plateau at higher concentration (more than 100 *µ*g/ml). The optimal working concentration of coating MOR hapten was the inflection point of titration curve. Ultimately, 50 *µ*g/ml was selected as the optimal MOR hapten concentration.

### 3.4. Incubation Time and Temperature of MOR Hapten Coating and Antigen-Antibody Reaction

Coating temperature and coating time were studied by a series of experimental data, including coated for 10 h at 4°C, coated for 12 h at 4°C, coated for 14 h at 4°C, coated for 1 h at 37°C, coated for 2 h at 37°C, coated for 3 h at 37°C, and coated for 4 h at 37°C. The results showed that MOR hapten coated for 12 h at 4°C had the highest fluorescence intensity in all conditions. Therefore, the plate coated 12 h at 4°C was recommended in this assay.

**Figure 6:**
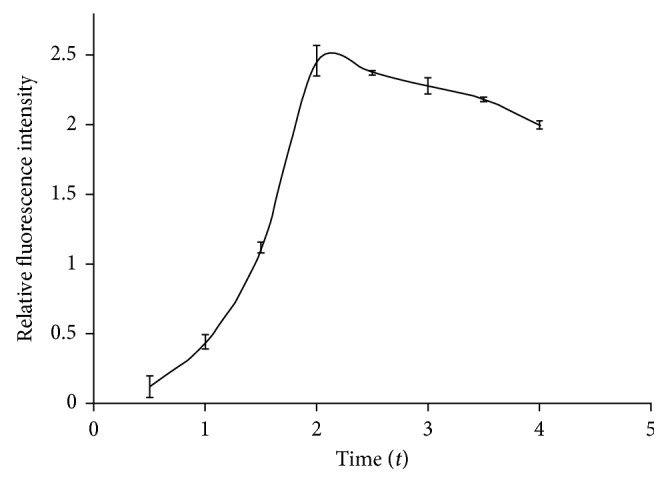
Incubation time curve for antigen-antibody reaction.

Incubation time and temperature are also important in the reaction of antigens and antibodies. In this study, two parts are involved. One is the binding reaction of MOR hapten coated on microporous plate with antibodies labeled FITC, and the other is the binding reaction of MOR as antigen in real samples with antibodies labeled FITC. Low temperatures could raise their binding fraction, and high temperatures could accelerate the reaction. Incubation time for antigen-antibody reactions was studied (shown in Figure 6), specifically from 0.5 h to 4 h at 37°C. The fluorescence intensity of the antigen-antibody reaction has been increasing rapidly until 2 h at 37°C, then strength slowly weakened. This result suggested that the optimal incubation time is 2 hours.

### 3.5. Influence of pH

**Figure 7:**
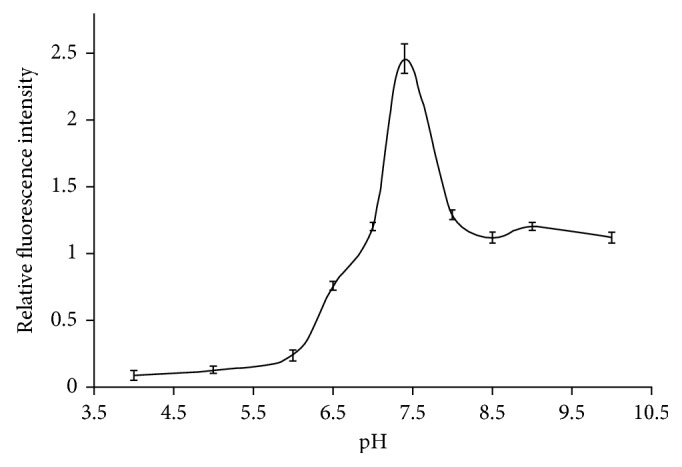
Curve of pH change in MOR competitive fluorescence immunoassays.

PBS buffer has widely used in immunoassays. Different pH values were tested from 4.0 to 10.0 and found the assay performed better in neutral media, as shown in Figure 7. The sensitivity was lower in both acidic and basic conditions. Therefore, the pH condition for the study was 7.4.

### 3.6. Ionic Strength

**Figure 8:**
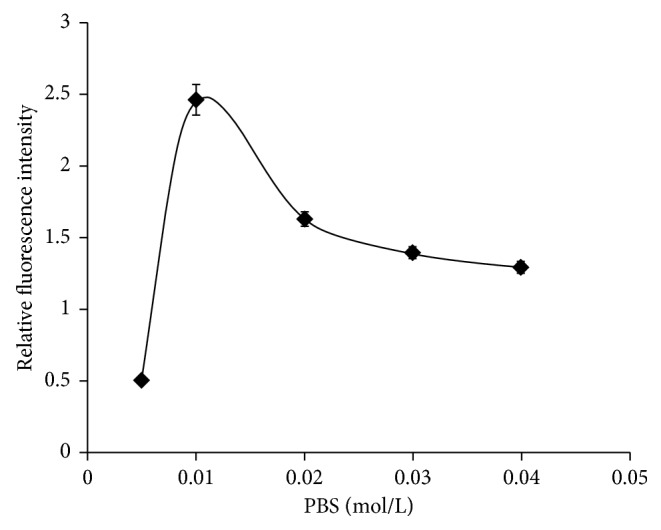
Curve of ionic strength change in MOR competitive fluorescence immunoassays.

Different salt concentrations of PBS, ranging between 0.5X and 4X of the original PBS buffer salt concentration, were examined (Figure 8). The results indicated that the fluorescence intensity increased sharply with the increase of buffer salt concentration before 0.01 mol/L. When the salt concentration was equal to 0.01 mol/L, it reached the highest and then decreased gradually. In the experiment, the concentration of 0.01 mol/L phosphate was the best buffer.

### 3.7. Fluorescence Competitive Curve and Calibration

**Figure 9:**
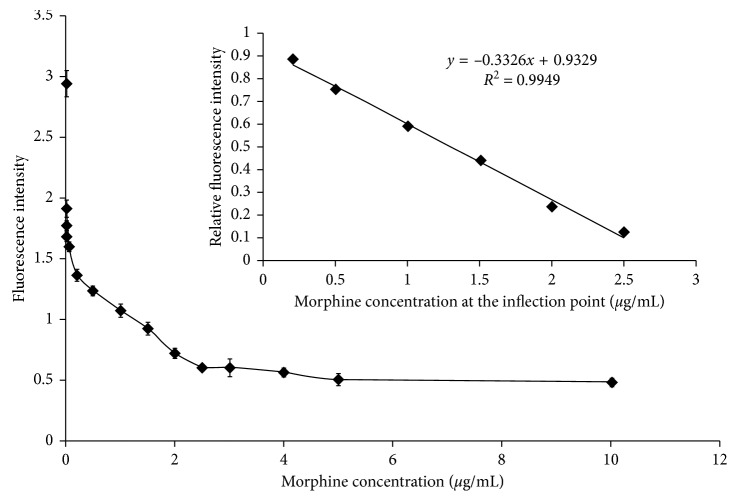
Competition curve of MOR, and MOR standard quantitative curve (insert).

The fluorescence competitive curve for MOR with different concentrations was obtained under optimized conditions. FITC-labeled antibody mixed with various MOR standard solutions (0, 0.001, 0.005, 0.01, 0.05, 0.2, 0.5, 1, 1.5, 2, 2.5, 3, 4, 5, and 10 *μ*g/mL, respectively) were inoculated on the MOR hapten-coated plates (50 *µ*g/ml). The fluorescence values and fluorescence competitive curve for MOR standard solutions with concentrations ranging from 0 to 10 *μ*g/mL are shown in Figure 9. In this curve, the *x*-axis is the concentration of MOR. The *y*-axis is the average value of five time's measurements. From the titration curve, the IC_50_ value (the concentration of the chemical used to reduce fluorescence by 50%) of 0.497 *μ*g/mL was measured. The calibration curve was analyzed for inflexion of fluorescence competitive curve, and MOR determination was done by plotting the relative fluorescence intensity curve based on the concentration of MOR. The curve has a linear response ranging from 0.2–2.5 *μ*g/mL. The linear regression curve was *y* = −0.3326*x* + 0.9329, where *y* is the relative fluorescence intensity (*F*–*F*_0_) and *x* is the concentration of MOR. The coefficient of correlation degree (*R*^2^) is 0.9949. The limit of detection was calculated to be 1 ng/mL (Figure 9 inset).

Most detection techniques serviced for determination of MOR in urine had been based on gas chromatography coupled to simple quadrupole mass spectrometry (GC-MS) with electron ionization [12–15]. However, conventional analytical instruments usually have weak recognition capabilities and limited accuracy when dealing with samples containing trace levels of MOR. In recent years, gas chromatography-tandem mass spectrometry (GC-MS/MS) technology has developed rapidly, providing higher potential for targeted analysis in terms of recognition and accuracy. As seen on Table 1, data acquired by GC-MS/MS method were compared to results obtained by the new competitive fluorescence immunoassay method. Although the linear range of MOR detection using the new method is narrower than measuring range of the GC-MS/MS, the detection limit of the new method is two times lower than that of the GC-MS/MS. Standard addition results of MOR in three different concentrations (low, middle, and high) of quantitative curves in urine samples can be seen from Table 2. The recovery and reproducibility of the new method is better than that of the GC-MS/MS method, and the RSD value was lower than 10%. In addition, sample pretreatment for the GC-MS/MS method is complex and time-consuming. In comparison, the new method dispenses with sample pretreatment, thus the new method is more ideal.

### 3.8. Immunoassay Specificity

In order to study the selectivity of new immunoassay, drug analogues with similar molecular structure including 6-acetylmorphine, 3-acetylmorphine, heroine, codeine, cocaine, ketamine, and ephedrine were evaluated for their potential cross-reactivity (CR). CR is used as a parameter to evaluate the selectivity of immunoassay and is usually calculated based on the IC_50_ value in the competition curve of each drug analogues. These seven drugs were also commonly abused like MOR and are thus likely to also be present in urine from drug abusers. In the experiment, these drugs were dissolved in ethanol and prepared as mentioned above. The CR rate was the ratio of IC_50_ value of MOR to that of coexisting drugs.

Results obtained under optimum conditions are summarized in Table 3. 6-Acetylmorphine had the highest CR rate with MOR, followed by 3-acetylmorphine. However, heroine, codeine, cocaine, ketamine, and ephedrine exhibited negligible CR rate. The data indicate that the selectivity of antibodies is based on the degree of molecular structure similarity. All the CR values of the other seven drugs were below 10%, and the antibody bound to MOR with the greatest affinity. The results suggested the MOR antibody prepared in our experiment has the highest specificity compared to others found in the literature. Susan et al. reported a series of monoclonal antibodies against MOR, in which CR values for codeine, apomorphine, heroin, papaverine, naloxone, and natrexone were 100%, 16.5%, lower than 0.05%, lower than 0.05%, lower than 0.05%, and lower than 0.05%, respectively [34]. Compared to the specificity of MOR antibody in our experiment, the CR rate with codeine was 1.6% and heroine was lower than 0.05%. The results demonstrate the MOR antibody acquired in our experiment had better specificity. Matyas et al. made MOR antibody bound with 6-acetylmorphine, that had a CR rate of 20.1% and 0.8% with heroin, the lowest in six experiments of different animals immunized [35]. Compared with the specificity of MOR antibody in our experiment, the CR rate with 6-acetylmorphine was 8.8% and heroine was lower than 0.05%. The results demonstrate the MOR antibody acquired in our experiment had better specificity. Thus, the new competitive fluorescence immunoassay method developed using this antibody can ensure that MOR was detected exclusively without interference from other coexisting compounds.

### 3.9. Forensic Sample Analysis

The urine samples were collected from the Fujian HuaMin forensic center. Urine samples from people of different age and sex were used to perform this study and analyzed with the optimized immunoassay. The concentration of MOR in human urine decreased over time. The concentration of MOR in urine obtained from MOR abusers is typically 1–100 *µ*g/mL under normal conditions [36, 37]. Using this new method, the samples were diluted 0–10 times as needed before being analyzed. Furthermore, the urine samples were also analyzed using the GC-MS/MS method. As seen in Table 4, the detection results of urine samples using the two different methods were almost the same. The relative deviations of results from these two different methods were lower than 10%. In conclusion, MOR in urine was detected with satisfactory results by the competitive fluorescence immunoassay method.

## 4. Conclusion

In this work, a competitive fluorescence immunoassay method for the determination of MOR in human urine has been described. In order to get the most accurate analysis results, various factors affecting the specific immunochemical interactions, such as reaction time, reaction temperature, environmental pH, and ionic strength, were investigated. Under the optimized conditions, the immunoassay yielded a detection limit of 1 ng/mL for MOR, with a working range of 0.2–2.5 *μ*g/mL. The detection limit for MOR in our new method is two times lower than conventional GC-MS/MS method. The immunoassay showed high selectivity to MOR and discriminated it against seven coexisting drugs that have similar molecular structures. Their CR rates were lower than 10%. The quantification results from our immunoassay had been verified by the GC-MS/MS method. Specifically, in the examination of MOR in urine samples, the relative deviation of six actual samples by these two methods is lower than 10%. Beneficial from simple, fast, sample-free pretreatment process, our method can be used to accurately detect MOR in human urine. Due to the existence of multifarious drug metabolites in human urine, simultaneous and multitarget testing is highly desirable. Since this assay is based on fluorescence detection, it can be easily integrated into microarray platform.

## Figures and Tables

**Table 1:** Linear regression equations, correlation coefficients, and limits of detection of MOR.

Method	Linear range (*μ*g/mL)	Linear regression equation	*R* ^2^	LOD (ng/mL)
Competitive fluorescence immunoassay	0.2–2.5	*y* = −0.3326*x* + 0.9329	0.9949	1
GC-MS/MS	0.1–50	*y* = 0.9401*x* − 194.9	0.9900	100

**Table 2:** Standard addition results of MOR in urine samples (*n*=5).

Detection method	Drug added (*µ*g/ml)	Detectable concentration of MOR	Recovery (%)	RSD (%)
Competitive fluorescence immunoassay	0.5	0.54893	109.79	1.0301
	1.5	1.5964	106.43	1.6171
	2.5	2.4971	99.884	3.4278

GC-MS/MS	0.1	0.10118	101.18	19.380
	12.5	11.075	88.600	8.0400
	50	38.770	77.540	6.2800

**Table 3:** Cross reactivity of MOR structurally similar compounds.

Coexisting substance	Molecule structure	CR rate (%)
MOR	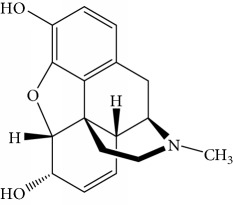	100
6-Acetylmorphine	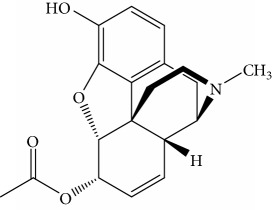	8.8
3-Acetylmorphine	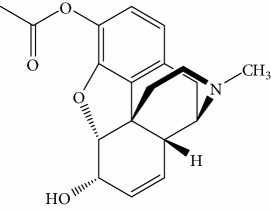	7.9
Codeine	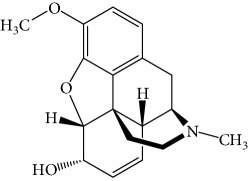	1.6
Heroine	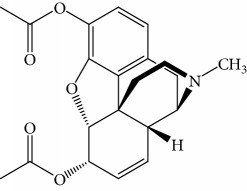	<0.05
Cocaine	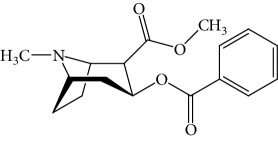	<0.05
Ketamine	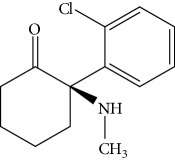	<0.05
Ephedrine	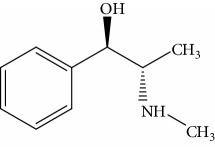	<0.05

**Table 4:** MOR concentrations in urine of medicolegal cases.

Sample code	Demographic		Quantitative results (*μ*g/mL)		Relative deviation (%)
	Age	Sex	Competitive fluorescence immunoassay	GC-MS/MS	
1	22	Female	5.286	5.691	7.12
2	28	Male	5.650	5.180	9.07
3	32	Female	10.64	11.03	3.54
4	35	Male	4.392	4.060	8.18
5	40	Male	13.80	12.95	6.56
6	45	Male	10.73	10.86	1.20

## Data Availability

The data used to support the findings of this study are included within the article.
